# Safeness of sodium fluorescein administration in neurosurgery: Case-report of an erroneous very high-dose administration and review of the literature

**DOI:** 10.1016/j.bas.2022.101703

**Published:** 2022-12-12

**Authors:** Francesco Restelli, Giulio Bonomo, Emanuele Monti, Giovanni Broggi, Francesco Acerbi, Morgan Broggi

**Affiliations:** Department of Neurosurgery, Fondazione IRCCS Istituto Neurologico Carlo Besta, Via Celoria, 11 20133, Milan, Italy

**Keywords:** Fluorescein, Glioma, Fluorescence, Fluorescein overdosage

## Abstract

**Introduction:**

Sodium Fluorescein has become a validated and widely used fluorescent dye in neuro-oncological surgery, thanks to its ability to accumulate in cerebral with a damaged blood–brain barrier. It concentrates at the tumor site, enhancing the lesion, and helps in the discrimination between tumor and normal brain parenchyma.

**Research question:**

This dye has a very well described profile of safeness, as a result of several applications in ophthalmology and, in recent years, also in neurosurgery. To date, no reviews are available on collateral effects of sodium fluorescein application in neurosurgery.

**Material and methods:**

The case of a young woman who underwent a potentially toxic dose (almost 3 ​g) of sodium fluroescein administration during anesthesia induction for a glioma surgery due to a medical error is presented, along with a review of available articles relates to collateral effects of sodium fluorescein in neurosurgery.

**Results:**

No toxic clinical phenomena occurred, and the microsurgical procedure was completed, achieving tumor gross total resection. Procedure resulted challenging due to an intense basal hyper-fluorescence, making difficult the visualization of brain tissues and the discrimination between normal brain and tumor.

**Discussion and conclusions:**

The good clinical and laboratory outcome of this patient further strengthens the idea that fluorescein-guided removal of brain tumors may be considered safe, beyond effective. By now, this is the first report of an erroneous so high dose administration of sodium fluorescein during a neurosurgical procedure and the first review of neurosurgical-reported collateral effects.

## Abbreviations

5-ALA: 5-Aminolevulinic Acid
AR: Adverse Reaction
BBB: Blood-Brain Barrier
CT: Computed Tomography
GTR: Gross Total Resection
HGG: High-grade Glioma
ICU: Neuro-Intensive Care Unit
MRI: Magnetic Resonance Imaging
SF: Sodium Fluorescein

## 1 Introduction

High-grade gliomas (HGGs) still represent the vast majority of malignant brain tumors, with a risible prognosis despite current standard treatments (Stupp et al., 2005). Nowadays, there is strong evidence supporting the extent of resection as a significant predictor of better survival in these patients (McGirt et al., 2009; Sanai and Berger, 2011). The use of fluorescence-guided tumor removal techniques has recently gained great interest, given the possibility to enhance tumor tissue during surgical operation. Among different fluorescent dyes, the use of the sodium salt of fluorescein (SF) has been explored, especially for HGGs surgery, thanks to its capacity to concentrate in areas of damaged blood-brain barrier (BBB) (Diaz et al., 2015).

The side effects of SF, since its first applications in general surgery and ophthalmology, have been extensively studied with few reports of severe complications and a generally safe profile (Kwiterovich et al., 1991; Marcus et al., 1984). This is particularly true in ophthalmology, where SF is being used nowadays as a valuable dye for retinal vessels study, resulting in its worldwide acceptance and uncountable consecutive applications in ophthalmic surgery (Yannuzzi et al., 1986; Kashani et al., 2017; Mendis et al., 2010). Looking at oncological and vascular neurosurgical applications, several authors claimed the totally safe profile of this dye and this constitutes, besides the proved ability to increase Gross Total Resection (GTR), one of the reason that largely contributed to its wide diffusion in neurosurgery in recent years (Diaz et al., 2015; Acerbi et al., 2018; Schebesch et al., 2016).

Here we present a case where a very high-dose of SF was erroneously administered during a glioma removal without severe adverse reactions (ARs) for the patient. Just few reports of SF collateral effects in neurosurgery have been described in literature and, to our knowledge, this is the first report of a so-high erroneous over-dosage of such dye in neurosurgical practice.

## 2 Case presentation

**Fig. 1:**
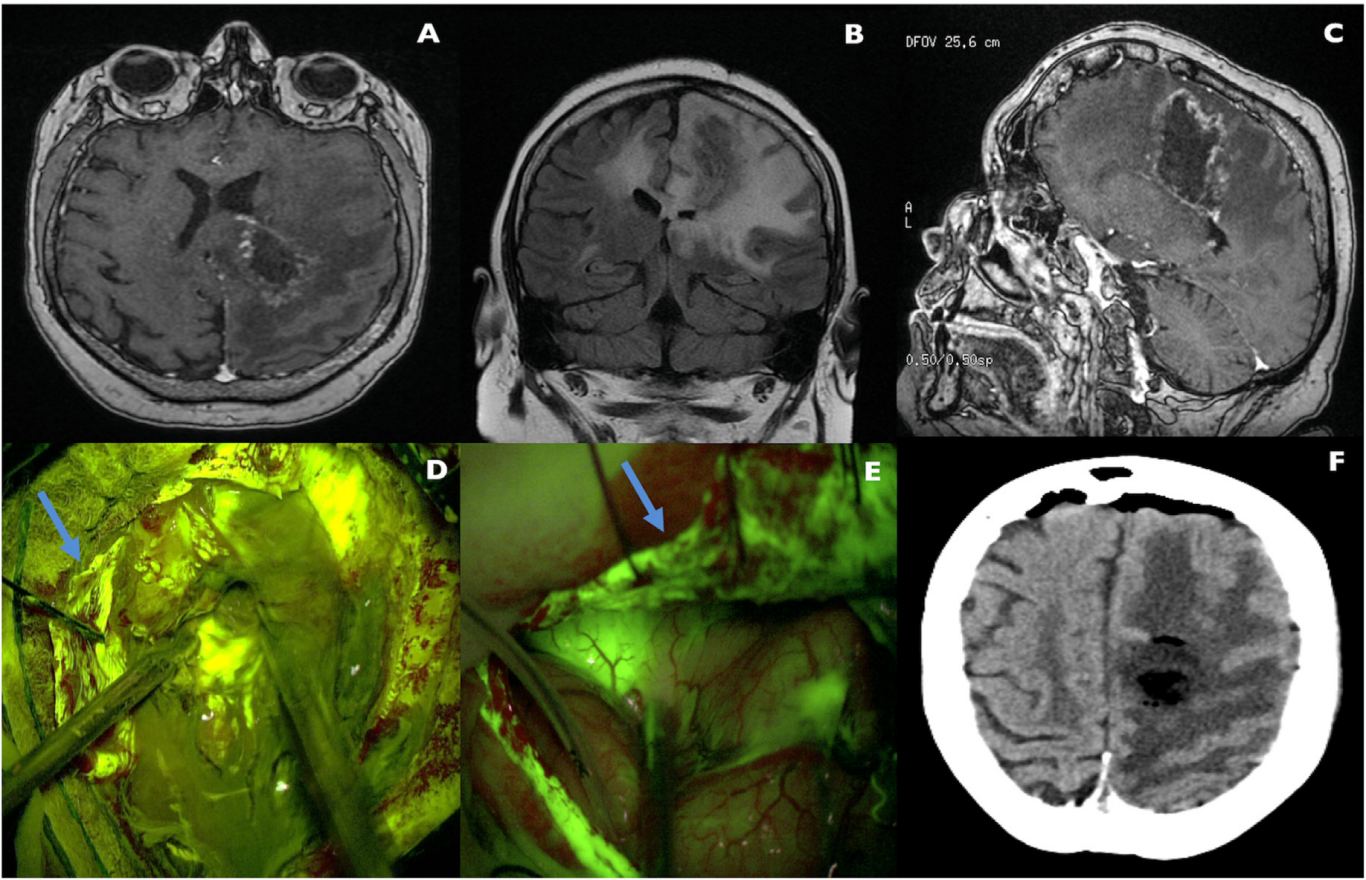
**Preoperative, intraoperative and postoperative images of the patient**. **A-C**. Preoperative axial T1 after contrast administration (A), coronal FLAIR (B) and sagittal T1 after contrast administration (C) MRI images of a left posterior frontal/para-trigonal HGG. As it can be seen, the ring-enhancing lesion is surrounded by diffuse brain edema. **D**. Intraoperative view during HGG removal with (very high dosage) SF-guided technique. The high basal fluorescence of the lesion and surrounding brain parenchyma (to note the high fluorescence intensity of dura, blue arrow) partially affected the clear discrimination between pathologic and healthy brain tissue. This aspect is clearly visible if the image in D is compared to a (normal dose) SF-guided technique removal of another HGG in a different patient (**E**), where tumor tissue is clearly different in fluorescence intensity if compared to normal brain parenchyma and dura mater fluorescence appears less intense (blue arrow in E). **F**. Postoperative CT scan disclosing the removal of the brain lesion, in absence of hemorrhagic complications. Unfortunately, we did not produce MRI images in the early postoperative period due to instrument breakdown. A postoperative MRI was taken in the rehabilitation clinic but, unfortunately, we were not able to obtain the images of the study. (For interpretation of the references to color in this figure legend, the reader is referred to the Web version of this article.)

A 34-year-old woman affected by generalized seizures started during pregnancy was diagnosed with a left posterior frontal glial lesion. After the induction of child-birth, a stereotactic frameless biopsy revealed an isocitrate dehydrogenase 1 positive and O6-methylguanine-DNA-methyltransferase promoter-mutated diffuse astrocytoma and the patient was therefore treated according to Stupp Protocol in another hospital. For disease progression, after few years the patient underwent a second-line treatment with Fotemustine, then followed by a third-line treatment with Temozolomide. At this time, the patient came to our attention, complaining mild expressive aphasia with a reduction in semantic and phonemic fluency, mild dysarthria, right hemiparesis and ataxic gait. A surgical excision of the lesion was then recommended and planned to be performed in microscopic view (Pentero900 – Carl Zeiss Meditec® Oberkochen, Germany) guided by SF technique with the aid of intraoperative motor evoked potentials monitoring, Magnetic Resonance (MR) and Computed Tomography (CT)-based neuro-navigation (BrainLab AG® Munich, Germany). Soon after patient intubation, a SF dose ten-times-bigger than recommended was erroneously injected to the patient due to an anesthesiologist mistake during the preparation of the drug (almost 30 mg/kg), resulting anyway in a GTR of the tumor (Fig. 1). Despite this event, no immediate or delayed clinical consequences occurred for the patient: no seizures, no relevant changes in lab exams, no clinical effects. The patient was then monitored in Neuro-Intensive Care Unit (ICU) till the following day, without complications, and then discharged after one week to rehabilitation clinic.

## 3 Discussion

Fluorescent agents are now widely used in neurosurgery. Among them, SF recently gained great interest in the neurosurgical community for oncological and neurovascular applications. In particular, its ability to accumulate in cerebral areas where a damage to the BBB has occurred allows the dye to become concentrated at tumor sites, making tumor tissue more clearly visible, particularly if a dedicated filter on the surgical microscope is available (Diaz et al., 2015; Acerbi et al., 2018). One of the main reason for the wide spread of SF is, besides its proved ability to increase GTR rates and a very affordable low cost (around 5 euros per vial), a well described safety profile, as confirmed by several years of application in general surgery and especially in ophthalmology (Yannuzzi et al., 1986; Acerbi et al., 2018; Kwan et al., 2006; Spaide et al., 2015).

Looking at the safety profile, most of reports of allergic reactions due to SF are related to angiographies for vitreo-retinal pathologies. These sporadic patients are generally reported to present mild allergic reactions, like nausea and vomiting, sneezing and pruritus, rather than severe, life-threatening ones, like laryngeal edema, seizures or circulatory shock. In 1984 a large study by Yannuzzi and colleagues deeply examined complications of fluorescein angiography through a survey among 2434 ophthalmologists, analyzing more than 220,000 exams. In this work, a total of 87% physicians reported a less-than 5% frequency of mild ARs, while only 2% described a frequency greater than 10%; only one death case directly related to SF injection was reported (Yannuzzi et al., 1986). In 2006, physicians at the Lion Eye Institute in Australia retrospectively reviewed 11898 fluorescein angiograms performed at their institute with only 132 ARs recorded, with no serious ones or deaths (Kwan et al., 2006). Also in another major work by Beleña et al. a 1,28% of ARs to fluorescein angiography out of 14455 exams was noticed, with just one case of bronchospasm as severe ARs and with nausea as the most common clinical presentation (Beleña et al., 2013). Severe adverse events remained very rare also in other large scale international studies (Katayama et al., 1990).

To our knowledge, just 2 cases of severe ARs by SF administration in neurosurgery are reported in the English literature (Tanahashi et al., 2006; Dilek et al., 2011). Table 1 summarizes the peculiarities of such cases. In the first one, a 74-year-old woman experimented severe hypotension with elevated serum Tryptase levels, as occurs in anaphylaxis or anaphylactoid reactions, around 20 ​min after a 20 mg/kg SF injection during a HGG resection (Tanahashi et al., 2006). The second case presented a 54-year-old man that suffered an anaphylactic reaction with severe hypotension, bradycardia, bilateral arms flushing and elevated serum levels of immunoglobulin E, right after a high-dose SF administration (20mg/Kg) (Dilek et al., 2011). In both cases patients had to be admitted to ICU, fully recovering after the administration of adrenalin, prednisolone, and atropine; both groups described the early and transient yellow discoloration of the skin and the urine due to quick renal excretion of the fluorescent dye.

**Table 1:** **Characteristics of reported patients with severe adverse reactions by SF administration during neurosurgical procedures.** (BP: Blood Pressure; bpm: beats per minute; GCS: Glasgow Coma Scale; HR: heart rate; y/o: years-old; IV: intravenous).

Reference	Gender/Age/Weight	Comorbidities	Site and type of lesion	Previous allergic reactions	Dosage – time of injection	Side effects	Lab Values	Management	Outcome	Previous reported adverse events by the Authors
Tanahashi et al. (Tanahashi et al., 2006)	Female, 74 y/o, no weight reported	Hypertension	Probably Glioma/unspecified location	No	20mg/Kg – after dural opening (no YELLOW560 filter reported)	After few minutes: - Severe hypotension - Mild tachycardia	- Elevated Tryptase level (14.4 ng/mL) - Elevated Histamine level (4.12 ng/mL)	- IV ephedrine 20mg - IV epinephrine 0.1mg - IV methylpredinosolone 500mg	Rapid recovery of cardiocirculatory status	not reported
Dilek et al. (Dilek et al., 2011)	Male, 54 y/o, 70Kg	Hypertension (on Telmisartan)	Glioma/temporal lobe	No	20mg/Kg – after dural opening (absence of YELLOW560 filter)	After few minutes: - Decrese in BP of 30/20 ​mm Hg - HR at 40 bpm - Flushing on arms	- Elevated IgE level (332U-upper limit at 100U) - Elevated Tryptase level (3.12 mg/dL)	Discontinuation of surgery plus: - IV adrenaline 1mg - IV prednisolone 100mg - IV atropine 1mg - colloid replacement - dopamine infusion (15 μg/kg per minute)	GCS 15 after some days, returned to neurosurgical service	0/121 patients between 2003 and 2010

Such reactions to SF may be due to multiple reasons but the exact pathogenic and immune mechanism is still unknown. Some possible mechanisms have been proposed such as vaso-vagal reactions, drug-related immediate hypersensitivity responses, vasospasm responses to the dye, histamine release of a non-allergic nature, anxiety-related sympathetic responses and contaminants in the drug (Yannuzzi et al., 1986). Some Authors also proposed that other comorbidities of patients, such as cardiovascular diseases, hypertension and diabetes mellitus, may facilitate these adverse events, considering such side effects not real reactions to the dye itself (Musa et al., 2006). Both the neurosurgical patients above described suffered from hypertension, strengthening the idea that such ARs may be facilitated by other medical conditions that ultimately favor the side effect manifestation.

The fact that in neurosurgical literature we couldn't find structured reports of side effects other than isolated severe ARs reports may be due to unidentified cases but also to unreported events. Nevertheless, almost every study where SF was used in neurosurgery, either for oncological or neurovascular cases, has always underlined the totally safe profile of this dye, even for high doses. Looking at this aspect, it must be kept in mind that in recent years the development of specific filters for surgical microscopes (Pentero with YELLOW560) allowed a reduction in SF doses necessary to enhance tumor tissue during oncological surgeries from 10 to 15 mg/Kg to less than 5mg/Kg (usually 3mg/Kg) (Acerbi et al., 2014, 2018). To the best of our knowledge, no serious ARs have been described in previous series published in literature on the use of SF in neurosurgery, even with doses of 20 mg/kg (Dallapiazza et al., 2014).

For the case described above, we used a Pentero Microscope with a YELLOW560 filter hardware, and our protocol consisted in administrating a dose of 3 mg/Kg of SF at the moment of patient intubation, therefore approximately 1 ​h before dural opening, following indication previously proposed by our group (Acerbi et al., 2015, 2018). We are strongly convinced of the appropriateness of this particular timing, given that, if serious adverse events such as anaphylactic reactions occur, they often become visible in few minutes after the administration of the dye, allowing anesthesiologists to manage them, before craniotomy. In the case here described, the patient received almost 3 ​g of SF few minutes after intubation, not manifesting any intraoperative or postoperative clinical or laboratory exam abnormalities, except from a very bright and fluorescent skin and yellow fluorescent urines, that persisted for approximately 24 hours after surgery. To note, we registered serious difficulties in visualization of brain tissues, and in discriminating between normal brain and tumor, due to intense basal hyper-fluorescence, visible also without activation of the specific microscope filter. Eventually, this operative mistake fortunately did not preclude the achievement of a GTR.

Far from us to put this single case-description among large ophthalmology case-series advocating SF safeness; the present report has the objective to describe a singular situation, to our knowledge never described before in neurosurgical literature, where almost 3 ​g of SF were given to a patient, not affecting the clinical status, presenting also a brief review on the topic.

## 4 Conclusions

SF is becoming a validated and widely used fluorescent dye in neurosurgery, with a very well described profile of safeness. A case of an erroneous very high-dose administration of this dye during a glioma removal surgery is here presented and the total absence of peri-operative abnormalities in clinical and serum analytical conditions of the patient further strengthens the idea that SF administration is a safe procedure and that may be considered routinely in oncological and neurovascular surgeries.

## Funding

This research was partially supported by the Associazione Paolo Zorzi per le Neuroscienze Onlus and by the Italian Ministry of Health (RRC).

## Declaration of competing interest

The authors declare that they have no known competing financial interests or personal relationships that could have appeared to influence the work reported in this paper.

## References

[bib1] Acerbi F., Broggi M., Eoli M., Anghileri E., Cavallo C., Boffano C., Cordella R., Cuppini L., Pollo B., Schiariti M., Visintini S., Orsi C., La Corte E., Broggi G., Ferroli P. (2014). Is fluorescein-guided technique able to help in resection of high-grade gliomas?. Neurosurg. Focus.

[bib2] Acerbi F., Broggi M., Broggi G., Ferroli P. (2015). What is the best timing for fluorescein injection during surgical removal of high-grade gliomas?. Acta Neurochir..

[bib3] Acerbi F., Broggi M., Schebesch K.-M., Höhne J., Cavallo C., De Laurentis C., Eoli M., Anghileri E., Servida M., Boffano C., Pollo B., Schiariti M., Visintini S., Montomoli C., Bosio L., La Corte E., Broggi G., Brawanski A., Ferroli P. (2018). Fluorescein-guided surgery for resection of high-grade gliomas: a multicentric prospective phase II study (FLUOGLIO). Clin. Cancer Res..

[bib4] Beleña J.M., Núñez M., Rodríguez M. (2013). Adverse reactions due to fluorescein during retinal angiography. JSM Ophtalmol.

[bib5] Dallapiazza R., Bond A.E., Grober Y., Louis R.G., Payne S.C., Oldfield E.H., Jane J.A. (2014). Retrospective analysis of a concurrent series of microscopic versus endoscopic transsphenoidal surgeries for Knosp Grades 0–2 nonfunctioning pituitary macroadenomas at a single institution. J. Neurosurg..

[bib6] Diaz R.J., Dios R.R., Hattab E.M., Burrell K., Rakopoulos P., Sabha N., Hawkins C., Zadeh G., Rutka J.T., Cohen-Gadol A.A. (2015). Study of the biodistribution of fluorescein in glioma-infiltrated mouse brain and histopathological correlation of intraoperative findings in high-grade gliomas resected under fluorescein fluorescence guidance. J. Neurosurg..

[bib7] Dilek O., Ihsan A., Tulay H. (2011). Anaphylactic reaction after fluorescein sodium administration during intracranial surgery. J. Clin. Neurosci..

[bib8] Kashani A.H., Chen C.L., Gahm J.K., Zheng F., Richter G.M., Rosenfeld P.J., Shi Y., Wang R.K. (2017). Optical coherence tomography angiography: a comprehensive review of current methods and clinical applications. Prog. Retin. Eye Res..

[bib9] Katayama H., Yamaguchi K., Kozuka T., Takashima T., Seez P., Matsuura K. (1990). Adverse reactions to ionic and nonionic contrast media. A report from the Japanese Committee on the Safety of Contrast Media. Radiology.

[bib10] Kwan A.S.L., Barry C., McAllister I.L., Constable I. (2006). Fluorescein angiography and adverse drug reactions revisited: the Lions Eye experience. Clin. Exp. Ophthalmol..

[bib11] Kwiterovich K.A., Maguire M.G., Murphy R.P., Schachat A.P., Bressler N.M., Bressler S.B., Fine S.L. (1991). Frequency of adverse systemic reactions after fluorescein angiography: results of a prospective study. Ophthalmology.

[bib12] Marcus D.F., Bovino J.A., Williams D. (1984). Adverse reactions during intravenous fluorescein angiography. Arch. Ophthalmol..

[bib13] McGirt M.J., Chaichana K.L., Gathinji M., Attenello F.J., Than K., Olivi A., Weingart J.D., Brem H., Quiñones-Hinojosa A.R. (2009). Independent association of extent of resection with survival in patients with malignant brain astrocytoma. J. Neurosurg..

[bib14] Mendis K.R., Balaratnasingam C., Yu P., Barry C.J., McAllister I.L., Cringle S.J., yi Yu D. (2010). Correlation of histologic and clinical images to determine the diagnostic value of fluorescein angiography for studying retinal capillary detail. Investig. Ophthalmol. Vis. Sci..

[bib15] Musa F., Muen W.J., Hancock R., Clark D. (2006). Adverse effects of fluorescein angiography in hypertensive and elderly patients. Acta Ophtalmol. Scand..

[bib16] Sanai N., Berger M.S. (2011). Extent of resection influences outcomes for patients with gliomas. Rev. Neurol..

[bib17] Schebesch K.M., Brawanski A., Hohenberger C., Höhne J. (2016). Fluorescein sodium-guided surgery of malignant brain tumors: history, current concepts, and future projects. Turk. Neurosurg..

[bib18] Spaide R.F., Klancnik J.M., Cooney M.J. (2015). Retinal vascular layers imaged by fluorescein angiography and optical coherence tomography angiography. JAMA Ophthalmol.

[bib19] Stupp R., Mason W.P., van den Bent M.J., Weller M., Fisher B., Taphoorn M.J., Belanger K., Brandes A.A., Marosi C., Bogdahn U. (2005). Radiotherapy plus concomitant and adjuvant temozolomide for glioblastoma. N. Engl. J. Med..

[bib20] Tanahashi S., Iida H., Dohi S. (2006). An anaphylactoid reaction after administration of fluorescein sodium during neurosurgery. Anesth. Analg..

[bib21] Yannuzzi L.A., Rohrer K.T., Tindel L.J., Sobel R.S., Costanza M.A., Shields W., Zang E. (1986). Fluorescein angiography complication survey. Ophthalmology.

